# Public Perception in Saudi Arabia Toward Herpes Zoster and Its Vaccination: A Cross-Sectional Study

**DOI:** 10.7759/cureus.58360

**Published:** 2024-04-16

**Authors:** Mohammad S AlKhowailed, Hatim M Alotaibi, Amwaj S Aljurays, Reem A Mohammad, Ghadeer M Alqahtani, Waleed Al Abdulmonem, Ahmed Alhumidi, Homaidan T Alhomaidan, Fuhaid M Alqossayir

**Affiliations:** 1 Department of Dermatology, College of Medicine, Qassim University, Buraydah, SAU; 2 Department of Medicine, Riyadh Third Health Culster, Ad-Dawadmi, SAU; 3 Department of Pathology, College of Medicine, Qassim University, Buraydah, SAU; 4 Department of Pathology, King Saud University, Riyadh, SAU; 5 Department of Family and Community Medicine, College of Medicine, Qassim University, Buraydah, SAU

**Keywords:** herpes zoster, shingels, knowledge, attitude, practice, vaccines, vaccination, saudi arabia

## Abstract

Background

Herpes zoster (HZ) or shingles, arises from the reactivation of the varicella zoster virus (VZV), mainly affecting older and immunocompromised individuals. Despite the efficacy of vaccines, vaccination rates in Saudi Arabia are low. Thus, this study aimed to assess the knowledge, attitude, and practice of the Saudi Arabian population toward HZ and its vaccination.

Methods

An observational cross-sectional study was carried out to evaluate the public perception in Saudi Arabia toward HZ and its vaccination, during the period from January to March 2024. Participants were selected using a non-probability, convenience sampling method, with recruitment facilitated through WhatsApp, a messaging app. Data has been analyzed using the statistical software Statistical Package for Social Sciences (SPSS), version 26.0 (IBM Corp., Armonk, NY). A p-value of <0.05 has been used to report the statistical significance.

Results

The study's demographic profile included 1237 participants, predominantly younger than 30 years (65.5%), with a female majority (65.7%). Public knowledge about HZ was limited, only 29.6% of participants recognized the risk of HZ post-chickenpox. More than half of the participants were not aware that the vaccine is provided by the Saudi Ministry of Health (MOH) for certain groups. However, over 75% are willing to receive the HZ vaccine upon physician recommendation.

Conclusion

This study shows a general lack of awareness about HZ and its vaccination in Saudi Arabia, including misconceptions about vaccination availability, recommendations, and the disease's complications. Gender differences in attitude and interest highlight the potential for tailored educational campaigns. Addressing these issues is essential for improving vaccination rates and mitigating HZ's impact.

## Introduction

Herpes zoster (HZ), or shingles, arises when the dormant varicella zoster virus (VZV) is triggered years later after an initial chickenpox infection [1]. After reactivation of VZV in the sensory root ganglia due to changes in the immunity, HZ manifests as a unilateral multiple painful vesicular rashes spread along the affected dermatome innervated by its corresponding sensory ganglion [2,3]. 

HZ typically affects elderly and immunocompromised people, causing major distress due to complications such as postherpetic neuralgia (PHN) [4]. PHN is one of the major debilitating conditions affecting about 5-30% of HZ patients, resulting in chronic pain and a serious influence on the patient's quality of life [5]. 

Around the world, the incidence of HZ has risen in recent years. In the general population aged ≥50 years old, the cumulative incidence of HZ varied from 5.23 to 10.9 cases per 1,000 person-years and from 2.9 to 19.5 cases per 1,000 population [6]. There is limited research on the incidence and prevalence of HZ in Saudi Arabia. In one study conducted in Saudi Arabia, the reported seroprevalence of varicella was about 86% [7]. 

The recent rise in cases brings attention to the importance of managing the low vaccination rate, which is attributed to public beliefs and perceptions toward HZ and its vaccination [8]. Despite the fact that vaccination of HZ is available as a preventive measure for shingles, the vaccination rate in Saudi Arabia is still low, reaching around 5% of the target population [9,10]. Therefore, there is an urgent need for an in-depth assessment of this negative practice toward HZ vaccination. 

Studies have suggested that the reasons for vaccine hesitancy include insufficient information about the diseases, uncertainty about the safety and efficacy of the vaccine, and other cultural factors [8]. A few studies have suggested a limited awareness of HZ and its vaccination among the Saudi population. However, the magnitude is unknown [11,12]. 

Previous literature on the public perception in Saudi Arabia of HZ and its vaccination is scarce, and the magnitude of the problem is still unknown. Therefore, we conducted this cross-sectional study in Saudi Arabia to assess the knowledge, attitude, and practice of the population of Saudi Arabia toward HZ and its vaccination.

## Materials and methods

An observational cross-sectional study was carried out to evaluate the public perceptions toward HZ and its vaccination in Saudi Arabia, during the period January to March 2024. Participants were selected using a non-probability, convenience sampling method, with recruitment facilitated through WhatsApp, a messaging app that is widely used in Saudi Arabia. The method of data collection employed was a well-structured online questionnaire, which consisted of multiple-choice questions (MCQs). The inclusion criteria for the study were: i) citizens and non-citizens of Saudi Arabia, ii) all genders, and iii) those 18 years of age or older. The exclusion criteria were: i) not giving consent, and ii) illiteracy. The minimum required sample size was 384 participants, based on a 95% confidence level and a ±5% margin of error, with an estimated proportion of 50% to approximate the large size of the target population. Since we used convenience sampling techniques, we increased the sample size to minimize bias and increase precision.

The data collection tool was a structured online questionnaire, divided into four different sections. The questionnaire was adapted from previous studies [13,14]. It was designed to gather comprehensive insights into public perceptions of HZ (shingles) and its vaccination in Saudi Arabia. The first section collected demographic information through seven questions, capturing details such as age, gender, educational background, employment status, and disease history. The following 14 questions in the second section aimed at assessing the participants’ knowledge regarding the symptoms, causes, and complications of HZ and vaccine eligibility criteria. The third section, with six questions, evaluated public attitudes toward HZ and its vaccination, including the level of understanding of the disease, its seriousness, and willingness to know more about disease prevention. Finally, a single question in the fourth section gauged the participants’ willingness to receive the shingles vaccine if recommended by a physician. 

Microsoft Excel software was used for data management. Data has been analyzed using the statistical software Statistical Package for Social Sciences (SPSS), version 26.0 (IBM Corp., Armonk, NY). Descriptive statistics (mean, standard deviation, frequencies, and percentages) have been used to describe the quantitative and categorical variables. Bivariate statistical analysis was carried out using the Student’s t-test to analyze the mean difference in the attitudes toward HZ and its vaccination between genders. A p-value of <0.05 has been used to report the statistical significance.

## Results

**Figure 1:**
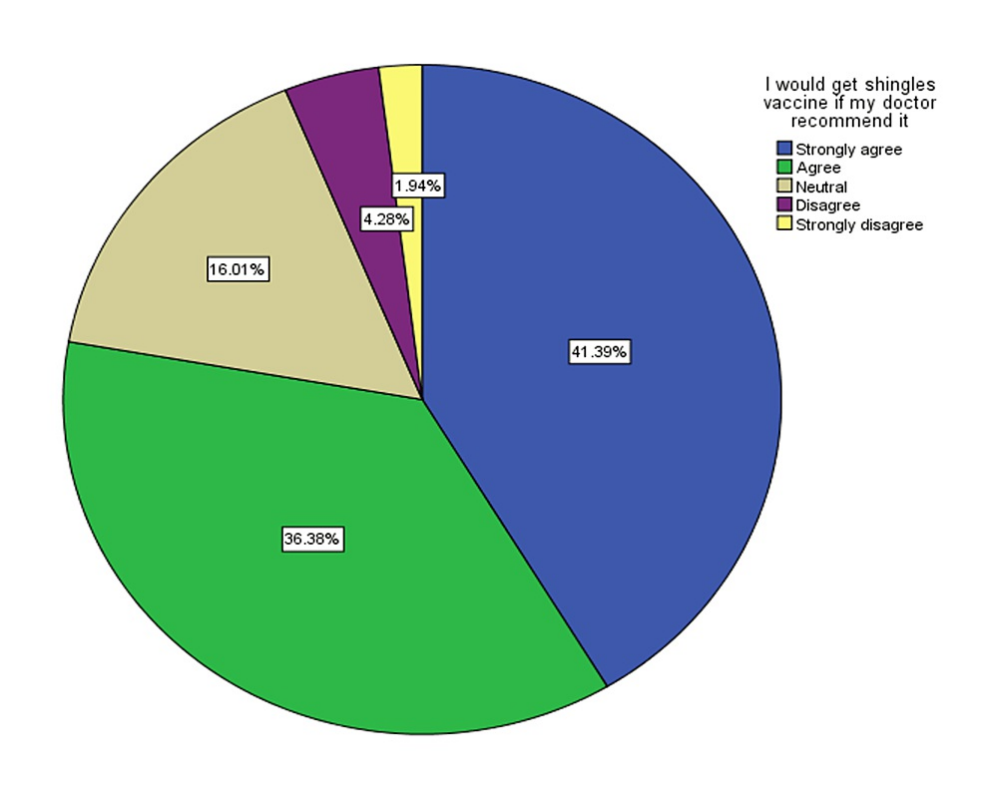
The willingness of the population in Saudi Arabia to get herpes zoster vaccine (n=1237)

The study's demographic profile included 1237 participants, predominantly younger than 30 years (65.5%), with a female majority (65.7%). Most participants were Saudi nationals (88.3%) and held a university degree or higher (72.3%). The study consisted mainly of students (46.4%), with a history of chickenpox reported by over half of the participants (50.8%), whereas a history of herpes zoster was relatively low (3.7%) (Table 1). Figure 1 illustrates a public willingness to get shingles vaccine if recommended.

**Table 1:** Demographic characteristics (n=1237)

Characteristics	n (%)
Age in years	
< 30 years old	810 (65.5)
≥ 30 years old	427 (34.5)
Gender	
Male	424 (34.3)
Female	813 (65.7)
Nationality	
Saudi	1092 (88.3)
Non-Saudi	145 (11.7)
Education	
High school or lesser	343 (27.7)
University or higher	894 (72.3)
Occupational status	
Student	574 (46.4)
Employed	362 (29.3)
Unemployed	301 (24.3)
History of chickenpox	
Yes	628 (50.8)
No	488 (39.5)
History of herpes zoster	
Yes	46 (3.7)
No	1100 (88.9)

Public knowledge surrounding HZ was limited, only 29.6% of participants recognized the risk of HZ post-chickenpox. However, uncertainty was prominent, with 59.1% unsure of the association. The awareness of the heightened risk for immunocompromised individuals was moderately recognized (57.6%). Symptoms such as rash (70.8%) and blisters (31.1%) were identified, yet there was less awareness of neuropathic pain (39.4%). About a third of the participants considered herpes zoster to be life-threatening (33.1%) (Table 2).

**Table 2:** Public knowledge about herpes zoster and its vaccination in Saudi Arabia (n=1237) HZ: Herpes zoster, MOH: Ministry of Health

Characteristics	n (%)
If an individual had chickenpox, he/she will be at risk of HZ	
Yes	366 (29.6)
No	140 (11.3)
Not sure	731 (59.1)
The lifetime risk of having HZ is up to one-third	
Yes	292 (23.6)
No	90 (7.3)
Not sure	855 (69.1)
Immunocompromised individuals are at a higher risk of HZ	
Yes	712 (57.6)
No	63 (5.1)
Not sure	462 (37.3)
Young people will not have HZ	
Yes	189 (15.3)
No	521 (42.1)
Not sure	527 (42.6)
If the rash of HZ circumvents the body, the individual will die	
Yes	205 (16.6)
No	322 (26.0)
Not sure	710 (57.4)
Individuals who have contact with HZ patients will acquire HZ	
Yes	404 (32.7)
No	352 (28.5)
Not sure	481 (38.9)
There are no drugs available for treating HZ	
Yes	124 (10.0)
No	619 (50.0)
Not sure	494 (39.9)
Symptoms of HZ include (not mutually exclusive)	
Rash	876 (70.8)
Blisters	385 (31.1)
Neuropathic pain	488 (39.4)
Blindness	85 (6.87)
Hearing loss	80 (6.4)
Do not know	245 (19.8)
Herpes zoster can be a life-threatening condition	
Yes	410 (33.1)
No	290 (23.4)
Not sure	537 (43.4)
HZ vaccine can reduce the incidence of disease by >50%	
Yes	662 (53.5)
No	36 (2.9)
Not sure	539 (43.6)
HZ vaccine can treat active HZ	
Yes	251 (20.3)
No	273 (22.1)
Not sure	713 (57.6)
Age group (in years) approved for vaccination against HZ is (not mutually exclusive)	
No age limit	508 (41.6)
< 18	131 (10.5)
18 - 50	201 (16.2)
≥18 years with a history of immunity condition	360 (29.1)
≥50	320 (25.8)
Eligible people to receive HZ vaccination include (not mutually exclusive)	
Did not have/unsure of the history of chickenpox	272 (21.9)
Had chickenpox, but no HZ	265 (21.4)
Had HZ before all of the above	81 (6.54) 356 (28.77)
Do not know	455 (36.7)
Saudi MOH provides herpes zoster vaccine for certain groups	
Yes	567 (45.8)
No	87 (7.0)
Not sure	583 (47.1)

Regarding vaccine eligibility, knowledge was relatively low. Only 25.8% correctly identified that the vaccine is recommended for individuals aged 50 and above, and 29.1% were aware that it is also recommended for those 18 years or older with a history of autoimmune disease or on immunosuppressive medication. When inquiring about the availability of the vaccine provided by the Saudi Ministry of Health (MOH), less than half (45.8%) were aware of its provision for certain groups, yet a significant 47.1% remained unsure (Table 2).

Table 3 highlights gender differences in attitudes toward HZ. Males displayed a marginally higher understanding of HZ, though not statistically significant. The perceived health impact of HZ was more pronounced among females (P = 0.002). Females also demonstrated a significantly greater interest in learning about HZ and its prevention measures (P = 0.001) for interest in learning about HZ and (P < 0.001) for interest in prevention).

**Table 3:** Comparison between males and females attitudes toward herpes zoster (HZ) and its vaccination in Saudi Arabia The attitude was measured using Likered scale questions where 1 = Strongly Agree, 2 = Agree, 3 = Neutral, 4 = Disagree, and 5 = Strongly Disagree. P-value of < 0.05 was used to report the statistically significant results.

Variable	Male; n=424 (Mean±SD)	Female; n=813 (Mean±SD)	P-value
I have an adequate understanding of HZ	3.40±1.184	3.45±1.096	0.405
HZ has a significant effect on health	2.36±.982	2.19±.851	0.002
I am worried about having HZ	2.86±1.149	2.89±1.134	0.577
I am interested in knowing more about HZ	2.09±1.029	1.90±.954	0.001
I have adequate channels in knowing how to prevent HZ	2.95±1.193	3.03±1.170	0.278
I am interested in knowing more about the prevention of HZ	2.13±1.048	1.87±.932	0.000

## Discussion

The findings of this study provide insight into the perceptions of the Saudi population toward herpes zoster and its vaccination. Our results showed that more than half of the respondents were not aware that the HZ vaccine was provided by the Saudi MOH. Conversely, a study performed in Al-Ahsa City showed that 87% of the participants knew that the HZ vaccine was available in Saudi Arabia [15]. This discrepancy can be attributed to the differences in sample size and the variation in healthcare availability and utilization across different regions of Saudi Arabia. 

Regarding the knowledge level of the vaccine's targeted groups in the present study, it was found to be limited, which is concerning given that the risk of HZ and its associated complications can be increased in certain groups [16]. Only a quarter of participants recognized that the vaccine is recommended for individuals aged 50 and above. Similarly, just under 30% were aware that the vaccine is also indicated for those 18 years or older with a history of autoimmune disease or who are on immunosuppressive medication. Our finding is in line with other research indicating a general lack of knowledge about vaccine indications among the public [12,15,17]. This gap in knowledge may be attributed to the absence of campaigns related to HZ and its vaccination. Therefore, targeted communication strategies to educate populations, particularly those who are at risk, about vaccine availability and eligibility should be enforced.

The current study found that participants underestimated the complications related to HZ. Similar findings were observed in a prior study [18]. This observation highlights the importance of educating the public on HZ and its impact, including postherpetic neuralgia and other long-term complications, as previous studies suggested that a higher level of knowledge about HZ and its vaccine could enhance the willingness of the population to get the vaccine [19,20].

Regarding the different attitudes toward HZ and its vaccination between genders, it was found that both genders have a similar level of self-perception toward HZ, but the female gender tends to have a more positive attitude toward learning about HZ and its prevention. This is similar to a previous study in which women had more positive attitudes toward HZ prevention and vaccination recommendations than men [21].

Furthermore, our findings suggest that participants' receptivity to vaccination is high upon physician recommendations. Similar results were found in previous research [14,22]. This is consistent with global evidence that healthcare professionals are trustworthy sources of vaccine-related information and play a central role in patients’ health decision-making [20,23]. 

Although there is limited knowledge among the Saudi population about HZ and its vaccine. There is a positive inclination toward vaccination against HZ upon physician recommendation. This highlights the importance of healthcare providers' engagement to educate the public in order to help increase awareness, increase vaccination rates, and thereby reduce the burden of HZ.

Limitations

Participants were selected using non-probability convenience sampling techniques. Also, non-citizens were part of the study. Thus, the results of this study cannot be generalized.

## Conclusions

This study shows a general lack of awareness about herpes zoster (HZ) and its vaccination in Saudi Arabia, including misconceptions about vaccination availability, recommendations, and the disease's complications. Gender differences in attitude and interest highlight the potential for tailored educational campaigns. Addressing these issues is essential for improving vaccination rates and mitigating the impact of HZ.
